# Activin B Stimulates Mouse Vibrissae Growth and Regulates Cell Proliferation and Cell Cycle Progression of Hair Matrix Cells through ERK Signaling

**DOI:** 10.3390/ijms20040853

**Published:** 2019-02-15

**Authors:** Pei Tang, Xueer Wang, Min Zhang, Simin Huang, Chuxi Lin, Fang Yan, Ying Deng, Lu Zhang, Lin Zhang

**Affiliations:** 1 Guangdong Provincial Key Laboratory of Tissue Construction and Detection, School of Basic Medical Sciences, Southern Medical University, Guangzhou 510515, China; tangpei29@126.com (P.T.); wyy85123@126.com (X.W.); zhangmin507@126.com (M.Z.); huangsimin2006@126.com (S.H.); 18402017785@163.com (C.L.); yanfang@smu.edu.cn (F.Y.); haruno_sakuring@163.com (Y.D.); 2 Guangdong Provincial Key Laboratory of Proteomics, Key Laboratory of Mental Health of the Ministry of Education, School of Basic Medical Sciences, Southern Medical University, Guangzhou 510515, China

**Keywords:** activin, vibrissae, hair matrix cell, cell proliferation, ERK

## Abstract

Activins and their receptors play important roles in the control of hair follicle morphogenesis, but their role in vibrissae follicle growth remains unclear. To investigate the effect of Activin B on vibrissae follicles, the anagen induction assay and an in vitro vibrissae culture system were constructed. Hematoxylin and eosin staining were performed to determine the hair cycle stages. The 5-ethynyl-2′-deoxyuridine (EdU) and Cell Counting Kit-8 (CCK-8) assays were used to examine the cell proliferation. Flow cytometry was used to detect the cell cycle phase. Inhibitors and Western blot analysis were used to investigate the signaling pathway induced by Activin B. As a result, we found that the vibrissae follicle growth was accelerated by 10 ng/mL Activin B in the anagen induction assay and in an organ culture model. 10 ng/mL Activin B promoted hair matrix cell proliferation in vivo and in vitro. Moreover, Activin B modulates hair matrix cell growth through the ERK–Elk1 signaling pathway, and Activin B accelerates hair matrix cell transition from the G1/G0 phase to the S phase through the ERK–Cyclin D1 signaling pathway. Taken together, these results demonstrated that Activin B may promote mouse vibrissae growth by stimulating hair matrix cell proliferation and cell cycle progression through ERK signaling.

## 1. Introduction

Hair has a wide range of functions including thermoregulation, sensory activity, and social interactions in mammals [1]. Mice display at least eight major hair types [1]. Vibrissae is a kind of hair, which is located on both sides of the nasal cavity and serves as a tactile sensory organ [2]. It has been extensively used in hair research as it is large in size and is free from hormonal influences [3]. 

The hair follicle (HF) is a skin appendage with a complex structure composed of hair bulbs, outer root sheaths (ORS), inner root sheaths (IRS), and the hair matrix [4].The HF undergoes life-long cyclic transformations between telogen (rest), anagen (growth), and catagen (regression) phases [5]. Every phase in HF cycling is stringently regulated by a variety of growth factors [6]. Activins are members of the transforming growth factor (TGF)-β superfamily [7]. Activin A (βAβA) and Activin B (βBβB) are the most common types expressed in various tissues [8]. The biological activities of activins are mediated by type I and type II receptors [9]. Some studies have shown that activins and their receptors play important roles in the control of HF morphogenesis and cycling [10]. Activin βA and its receptors are widely expressed in HFs [11]. Matzuk and colleagues generated Activin subunit βA knockout mice and found that the vibrissae follicles of the mutants showed delayed maturation [12]. Human dermal papilla (DP) spheres with Activin A knockdown show severely impaired HF neogenesis [13]. In our previous study, we found that Activin B can promote mouse pelage HF initiation and development [14]. In a further study, we showed that Activin B could promote HF regeneration after skin wound healing [15]. However, considering the substantial anatomical, biological, and regulatory differences between different types of HFs [16], the role of Activin B in the regulation of vibrissae follicle regeneration is still not clear. 

Hair matrix cells reside in the hair bulb [17]. Their proliferation is an important event for hair growth [18]. Some factors which suppress hair matrix cell proliferation can induce an imbalance of HF cycles, resulting in hair loss [19,20]. Nakamura et al. demonstrated that Activin B receptors are expressed in the hair matrix position [10]. However, the role of Activin B in hair matrix cell proliferation and its subsequent signaling pathway have not been reported.

The signaling mechanisms of Activin include Smad-dependent and Smad-independent pathways [21]. Some studies have demonstrated that activin activates mitogen-activated protein kinase (MAPK) pathways [22,23]. MAPKs, comprising JNK, P38, and ERK, are considered to play crucial roles in HF morphogenesis and regeneration [24]. The JNK signaling pathway has been reported to be involved in the apoptosis of HF cells in TGF-β induced premature catagen phase [25]. Epidermal growth factor (EGF) and its receptor were also shown to promote anagen phase via the ERK pathway [26].

In the present study, firstly, we investigated the function of Activin B in mouse vibrissae follicle growth by an anagen induction assay and an in vitro vibrissae culture system. Secondly, we assessed the effect of Activin B on the proliferation of hair matrix cells in vivo and in vitro. Thirdly, we investigated the regulation of the MAPK signaling pathway in hair matrix cell proliferation induced by Activin B. Lastly, we explored the role of Activin B in hair matrix cell cycle progression and the relative signaling in this process. Investigating the role and mechanisms of Activin B in hair growth may help to explore the new targets and theoretical options for hair regeneration.

## 2. Results

### 2.1. 10 ng/mL Activin B Accelerated Vibrissae Follicle Growth

**Figure 1:**
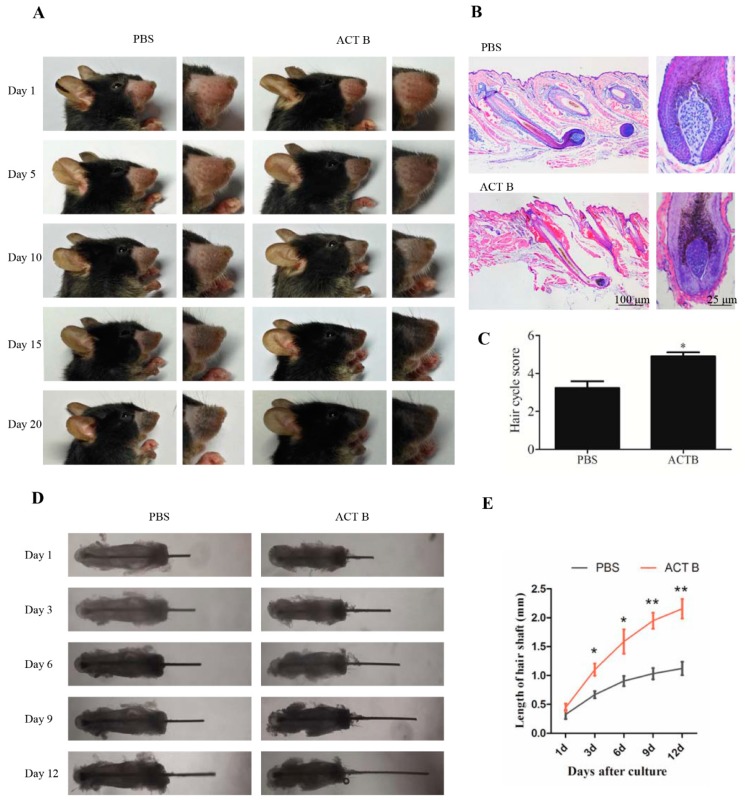
Activin B accelerated vibrissae follicle growth. (**A**) Gross images of mouse vibrissae treated with 10 ng/mL Activin B or phosphate buffer solution (PBS) for 1, 5, 10, 15, and 20 days in depilation-induced models. (**B**) Representative hematoxylin and eosin (H&E) staining images of vibrissae follicles at 10 days after treatment with PBS or 10 ng/mL Activin (ACT) B. (**C**) Hair cycle score for the anagen hair follicles in the PBS and ACT B groups 10 days after depilation, and twenty vibrissae follicles were collected from five mice in each group for quantitative evaluation. (**D**) Representative pictures of vibrissae cultured with or without 10 ng/mL Activin B in an in vitro vibrissae culture system. Pictures were taken on 1, 3, 6, 9, and 12 days after culture. (**E**) Growth curve of the vibrissae shafts over time in the two groups. * *p* < 0.05; ** *p* < 0.01, compared with the PBS group. Three independent experiments were conducted per data point. All error bars indicate SEM.

To determine whether Activin B stimulates vibrissae follicle regeneration in vivo, depilation was used to induce vibrissae follicles into a homogeneous anagen stage. We smeared 1 mL of 10 ng/mL Activin B or PBS on the vibrissae skin three times a day for 20 days, and observed the color of the vibrissae skin and the hair present every day (Figure 1A). After 10 days of depilation, vibrissae skin in the Activin B-treated mice turned black, and hair shafts emerged through the epidermis. In contrast, the vibrissae skin in the PBS group were gray at 10 days and turned to black at 15 days after depilation (Figure 1A). Owing to the strict coupling of follicular melanogenesis and HF cycling, the change in skin pigmentation is a characteristic of anagen development [27,28,29].

We then compared the hair cycle stages of vibrissae follicle in the two groups at 5 days and 10 days after depilation by morphological criteria using hematoxylin and eosin (H&E) staining, as described previously [30]. At 5 days after depilation, the vibrissae growth in the Activin B group was accelerated, with tips of the IRSs present and a larger DP than the PBS group (Figure S1). At 10 days after depilation, the vibrissae follicles extended into the dermis in the two groups. The hair shaft and IRS reached the hair canal in the Activin B-treated group (Figure 1B). Further, the bulb was enlarged, and the dermal papilla were narrowed in the Activin B group (Figure 1B). All these results suggest that vibrissae follicle treatment with Activin B causes them to enter anagen Stage 5. However, the vibrissae follicles in the PBS group were mostly in anagen Stage 3, with the characteristics that the DP is still of loose consistency and not fully surrounded by hair matrix cells (Figure 1B). The quantitative evaluation of hair follicles showed that the average anagen stages of hair follicles in the 10 ng/mL Activin B group were Stage 5.0 ± 0.21, significantly higher than those in the PBS groups with Stage 3.1 ± 0.35 (Figure 1C). Taken together, these results suggest that 10 ng/mL Activin B promotes vibrissae growth in vivo.

### 2.2. 10 ng/mL Activin B Promoted Vibrissae Hair Shaft Elongation in an Organ Culture Model

We next used an organ culture model to examine the effect of Activin B on the regulation of vibrissae follicle hair growth. In the gross images, the length of vibrissae in the Activin B group was longer than that in the PBS group (Figure 1D). The growth rate of the vibrissae hair shaft was approximately 0.12 ± 0.13 mm/day in the Activin B group but only 0.07 ± 0.06 mm/day in the PBS group (Figure 1E). These data also show that Activin B could promote vibrissae growth.

### 2.3. Activin B Enhanced the Proliferation of Hair Matrix Cells In Vivo and In Vitro

**Figure 2:**
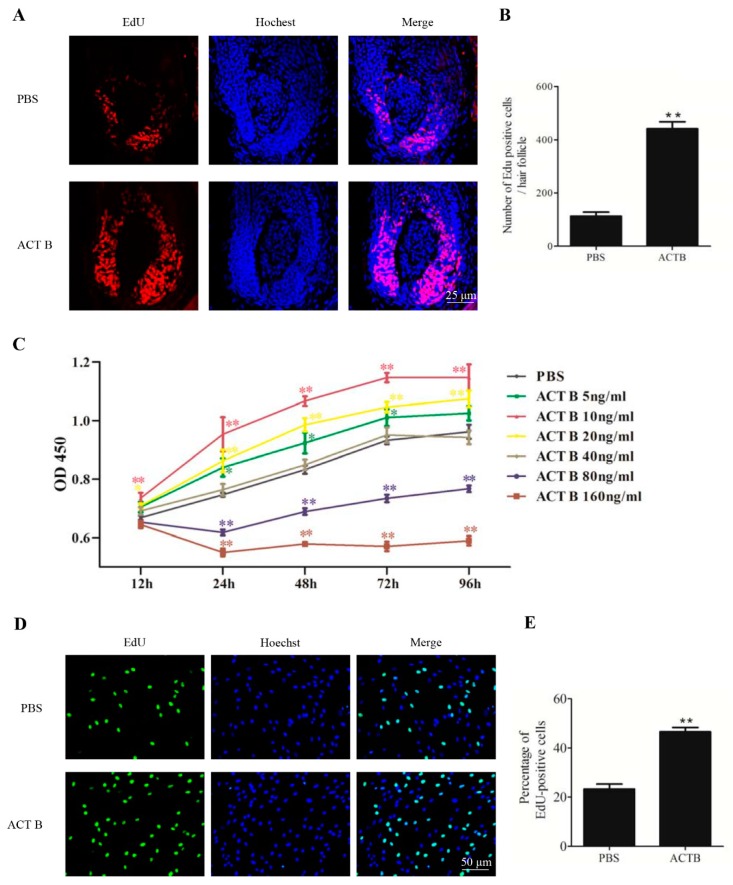
Activin B enhanced the proliferation of hair matrix cells in vivo and in vitro. (**A**) Representative images of EdU staining (red fluorescence) of vibrissae follicles treated with PBS or 10 ng/mL Activin B at 10 days after treatment. Nuclei were counterstained with Hoechst 33342 (blue fluorescence). (**B**) Ten vibrissae follicles per group were collected from five mice in each group and the number of EdU-positive cells in the two groups was counted. (**C**) Human hair germinal matrix cells (HHGMCs) were treated with Activin B at the concentration of 5, 10, 20, 40, 80, or 160 ng/mL. The CCK-8 assay was performed at 12, 24, 48, 72, and 96 h after treatment. (**D**) HHGMCs treated with 10 ng/mL Activin B or PBS were subjected to EdU assay (green fluorescence). Nuclei were counterstained with Hoechst 33342 (blue fluorescence). (**E**) The number of positive cells was counted manually in ten fields-of-view randomly selected from each well. The percentages of positive cells were calculated. * *p* < 0.05; ** *p* < 0.01, compared with the PBS group. Three independent experiments were conducted per data point. All error bars indicate SEM.

Hair matrix cell proliferation is required for HF formation [18]. We then assessed the effect of Activin B on hair matrix cell proliferation. The number of EdU-positive cells in the hair matrix was markedly increased in the Activin B group compared to that in PBS group at 10 days after treatment (Figure 2A,B), suggesting that Activin B promotes hair matrix cell proliferation in vivo. 

We next examined the effect of Activin B on the proliferation of hair matrix cells in vitro by CCK-8 and EdU assays. The CCK-8 cell growth assay showed that in the range of 5 ng/mL to 20 ng/mL, Activin B enhanced the proliferation rate of hair matrix cells, whereas at the concentrations of 80 ng/mL and 160 ng/mL, Activin B inhibited the proliferation of hair matrix cells (Figure 2C). Among the concentrations we tested, 10 ng/mL Activin B was most effective in promoting human hair germinal matrix cell (HHGMC) proliferation (Figure 2C). Consistent with this, the percentage of EdU-positive cells in the Activin B group (10 ng/mL) was much higher than that in the PBS group (Figure 2D,E).

The results of the in vivo and in vitro experiments indicate that 10 ng/mL Activin B enhances the proliferation of hair matrix cells.

### 2.4. Activin B Modulated the Proliferation of Hair Matrix Cells Through the ERK–Elk1 Signaling Pathway

**Figure 3:**
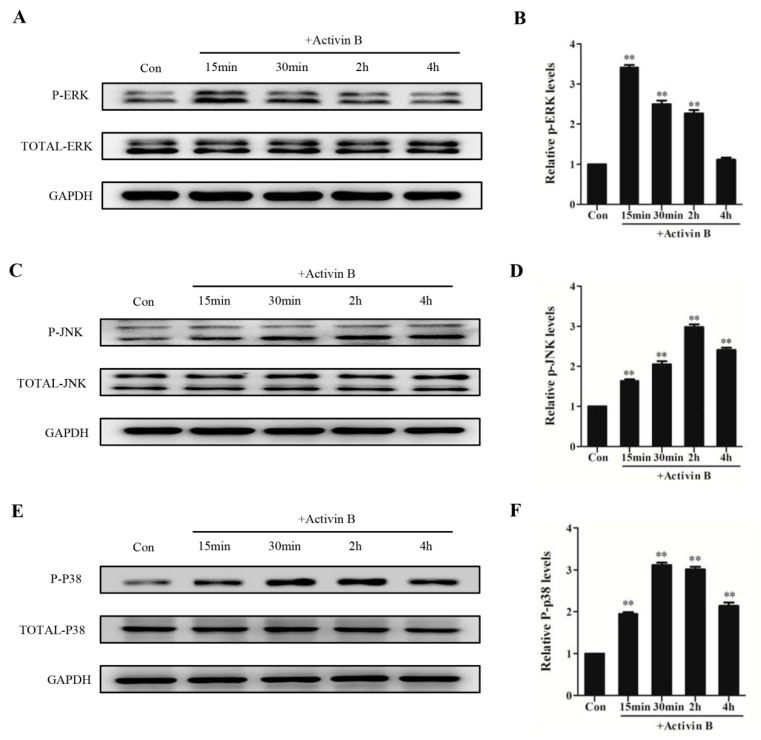
Activin B stimulated JNK, ERK, and P38 phosphorylation in hair matrix cells. (**A**,**B**) The plot and the relative quantification of the expression of phosphorylation of ERK in HHGMCs treated with 10 ng/mL Activin B for 15 min, 30 min, 2 h, and 4 h. (**C**,**D**) The plot and the relative quantification of the expression of phosphorylation of JNK in cells treated with 10 ng/mL Activin B for different times. (**E**,**F**) The plot and the relative quantification of the expression of phosphorylation of P38 in cells treated with 10 ng/mL Activin B for different times. * *p* < 0.05; ** *p* < 0.01, compared with the PBS group. Three independent experiments were conducted per data point. All error bars indicate SEM.

To examine whether the MAPK signaling pathway is involved in Activin B-induced hair matrix cell proliferation, we determined the phosphorylation status of ERK, JNK, and P38 in HHGMCs treated with Activin B. We found that the phosphorylated levels of ERK, JNK, and P38 were all obviously enhanced by Activin B. The level of ERK phosphorylation peaked at 15 min, and returned to the basal level at 4 h after incubation with Activin B (Figure 3A,B). The level of JNK phosphorylation was activated at 15 min and at its peak at 2 h (Figure 3C,D). The expression of phosphorylated P38 was significantly increased after 15 min of treatment and reached its peak at 30 min (Figure 3E,F). 

**Figure 4:**
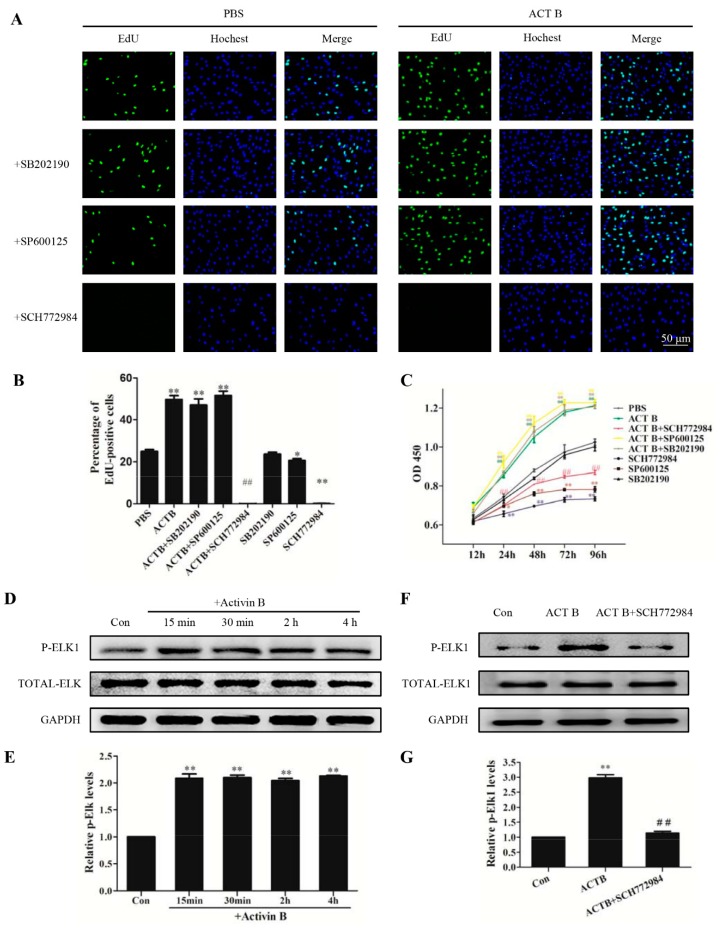
Activin B modulated the cell growth of HHGMCs through the ERK–Elk1 signaling pathway. (**A**) HHGMCs were serum starved for 24 h followed by treatment with SCH772984 (5 μM), SP600125 (5 μM), or SB202190 (5 μM) for 2 h. After treatment with PBS or 10 ng/mL Activin B for 48 h, EdU staining (green fluorescence) was performed. Nuclei were counterstained with Hoechst 33342 (blue fluorescence). (**B**) The percentage of EdU-positive cells was calculated. (**C**) For the CCK-8 assay, HHGMCs were pretreated as (**A**) described. The CCK-8 assay was conducted at 12, 24, 48, 72, and 96 h after Activin B treatment. (**D**,**E**) The plot and the relative quantification of the expression of phosphorylation of Elk1 in HHGMCs treated with 10 ng/mL Activin B for 15 min, 30 min, 2 h, or 4 h. (**F**,**G**) The plot and the relative quantification of the expression of phosphorylation of Elk1 in the cells pretreated with SCH772984. * *p* < 0.05; ** *p* < 0.01, compared with the PBS group. ^#^ *p* < 0.05; ^##^ *p* < 0.01, compared with Activin B group. Three independent experiments were conducted per data point. All error bars indicate SEM.

To determine whether ERK, JNK, and P38 signaling were involved in HHGMC proliferation induced by Activin B, JNK inhibitor SP600125, ERK inhibitor SCH772984, and P38 inhibitor SB202190 were used to pretreat the HHGMCs. We found that SCH772984 abolished the growth of HHGMCs with or without Activin B (Figure 4A,B). However, neither SB202190 nor SP600125 inhibited HHGMCs proliferation induced by Activin B (Figure 4A,B). These data suggest that ERK signaling, but not JNK or P38, was involved in Activin B-induced cell proliferation in HHGMCs. These results were also supported by the CCK-8 assay (Figure 4C). We next investigated the effects of SCH772984 on hair shaft elongation using an organ culture model, and found that SCH772984 can block vibrissae growth induced by Activin B (Figure S2).

Elk1 is a major downstream cytosolic transcription factor of ERK signaling. We found that phosphorylation of Elk1 was significantly increased at 15 min after treatment with Activin B (Figure 4D,E). However, SCH772984 inhibited Activin B-induced phosphorylation of Elk1 (Figure 4F,G). These results indicate that 10 ng/mL Activin B promote the proliferation of HHGMCs through the ERK–Elk1 signaling pathway.

### 2.5. Activin B Accelerated the Cell Cycle Transition of HHGMCs from the G0/G1 Phase to the S Phase Through the ERK–Cyclin D1 Pathway

**Figure 5:**
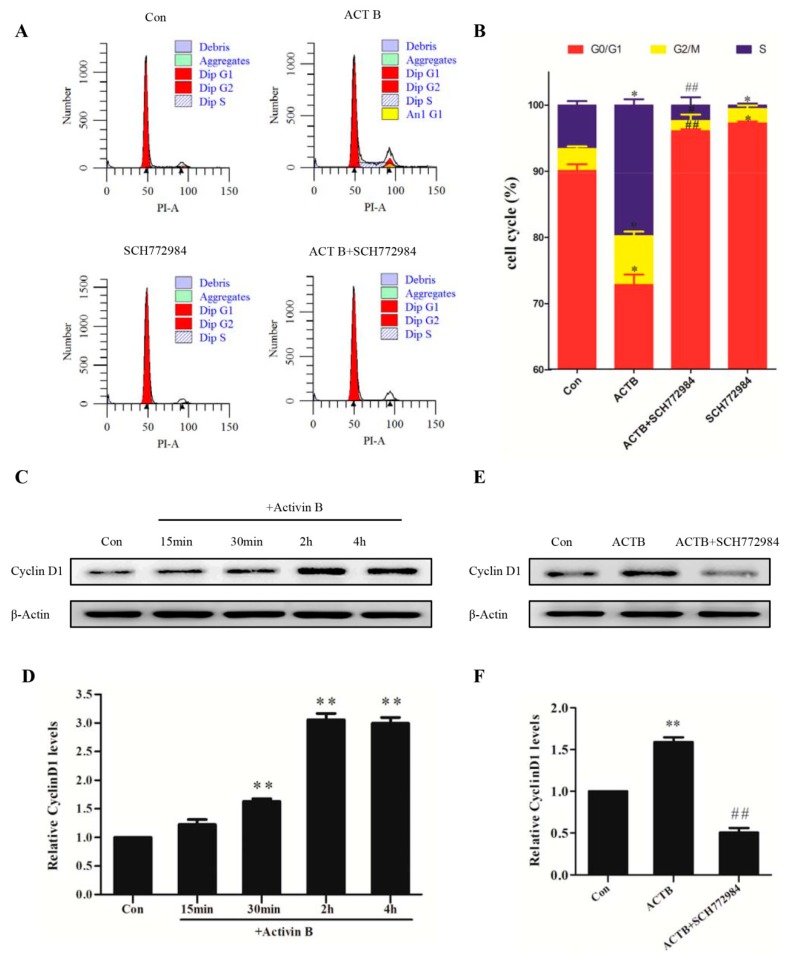
Activin B accelerated the cell cycle transition of HHGMCs from the G0/G1 phase to the S phase through the ERK–Cyclin D1 pathway. (**A**) HHGMCs were serum starved for 24 h and pretreated with or without SCH772984 (5 μM) for 2 h. After treatment with 10 ng/mL Activin B or PBS for 48 h, the cell cycle distribution was measured by flow cytometry using propidium iodide (PI) staining. (**B**) The percentages of cell cycle phase in G0/G1, S, and G2/M are shown in a bar graph form. (**C**,**D**) The plot and the relative quantification of the expression of Cyclin D1 in the cells treated with 10 ng/mL Activin B for 15 min, 30 min, 2 h, and 4 h. (**E**,**F**) The plot and the relative quantification of the expression of Cyclin D1 in the cells pretreated with SCH772984. * *p* < 0.05; ** *p* < 0.01, compared with the PBS group. ^#^ *p* < 0.05; ^##^ *p* < 0.01, compared with Activin B group. Three independent experiments were conducted per data point. All error bars indicate SEM.

We then tested the role of Activin B in the regulation of the cell cycle in HHGMCs by flow cytometry. The results showed that Activin B significantly decreased the percentage of cells in the G0/G1 phase and increased the cell population in the S phase (Figure 5A,B), which suggested that Activin B facilitated G1–S transition in the HHGMCs. However, the positive effect of Activin B on the cell cycle was blocked by ERK inhibitor SCH772984 (Figure 5A,B).

Cyclin D1 is a G1/S transition regulatory protein [31]. We found that the expression of Cyclin D1 was significantly increased by treatment with Activin B (Figure 5C,D). Furthermore, the upregulation of Cyclin D1 expression induced by Activin B was weakened by SCH772984 (Figure 5E,F). Taken together, these results indicate that 10 ng/mL Activin B promotes cell cycle progression from the G0/G1 phase to the S phase through the ERK–Cyclin D1 signaling pathway.

## 3. Discussion

In postnatal life, HFs are periodically regenerated through a continuous cycle that includes the telogen, anagen, and catagen stages [32]. The onset of anagen in HFs recapitulates HF growth and development [33]. In this study, our results demonstrated that Activin B promotes vibrissae growth and accelerates mouse vibrissae anagen progression. This is consistent with the results of our previous study that Activin B promotes the hair cycle of mouse pelage HFs [14]. These data suggest that Activin B plays a positive role in hair growth in different types of HFs. However, our finding is at odds with Jaenisch’s finding [34]. Jaenisch and colleagues found that Activin βB subunit gene disruption did not influence vibrissae HF development, suggesting that Activin B has little effect in vibrissae follicle development. However, Brown’s study indirectly demonstrated the potential role of Activin B in vibrissae development [35]. Considering that the redundancy of activins may hamper the investigation of the physiological role of Activin B in vibrissae development in knockout mice studies, this may explain the discrepancy between Jaenisch’s results and those of our study.

Normal HF growth depends on the activation of hair matrix cells [4]. We found that Activin B significantly increased the proliferation of hair matrix cells in vivo and in vitro. Considering that hair matrix cells are highly proliferated in the anagen phase and play a part in the progression of the hair cycle [36], the positive effect of Activin B on hair matrix cell proliferation may count towards the vibrissae growth. Our finding is consistent with the report that recombinant human fibroblast growth factor-20 stimulated the proliferation of hair matrix cells and induced vibrissae follicle growth [37]. Han et al. also found that CsA is involved in mouse vibrissae growth through promoting hair matrix cell activation [38]. 

Determining the molecular mechanism that controls hair growth is of great importance in hair research. The MAPK pathway is an essential intracellular signal transduction pathway that regulates cell proliferation [39]. In the present study, our data showed that Activin B promotes hair matrix cell proliferation through the ERK–Elk1 pathway. Meldrum et al. reported that metalloproteinase is involved in UVB radiation-induced G1-S cell cycle progression in keratinocytes by the ERK/AKT/CyclinD1 pathways [40]. Consistent with these findings, we showed that Activin B promotes G0/G1 to S phase transformation through the ERK–Cyclin D1 pathway. In addition, we found that the levels of AKT phosphorylation were significantly increased 2 h after treatment with Activin B (Figure S3), suggesting that AKT may also be a downstream signal of ERK during Activin B-induced G1–S cell cycle progression of hair matrix cells. The previous study showed that cell cycle progression in the G1 phase has a key role in cell proliferation [41]. This gives a clue that Activin B may contribute to a growth-promoting effect on HFs, through regulating the cell cycle checkpoint protein Cyclin D1 by the ERK pathway. 

**Figure 6:**
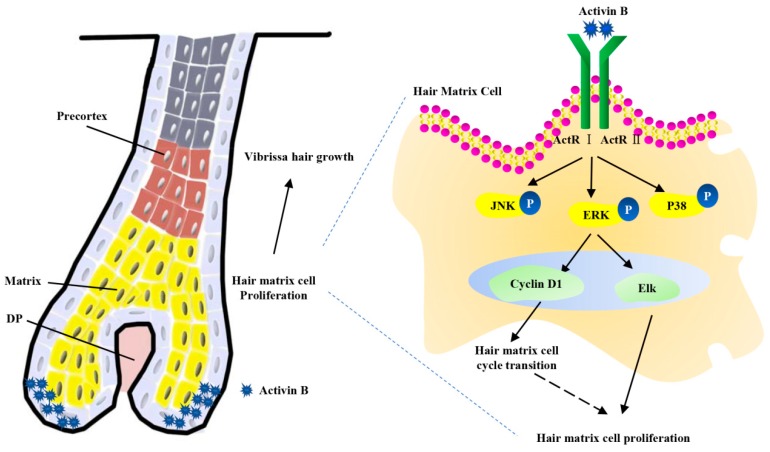
The proposed mechanisms model of Activin B on mouse vibrissae growth. Activin B accelerates vibrissae follicle growth. Activin B modulates the cell growth of hair matrix cells through the ERK–Elk1 signaling pathway and accelerates hair matrix cell transition from the G0/G1 phase to the S phase through the ERK-Cyclin D1 pathway.

In conclusion, our study demonstrated that 10 ng/mL Activin B accelerates vibrissae follicle growth in vitro in an organ culture model and in vivo in a mouse model. This is possibly mediated by enhancing hair matrix cell proliferation through the ERK–Elk1 signaling pathway and by accelerating hair matrix cell transition from the G0/G1 phase to the S phase through the ERK–Cyclin D1 pathway (Figure 6). Our findings may have clinical implications for hair loss treatment.

## 4. Materials and Methods

### 4.1. Mice

Seven-week-old male C57BL/6 mice were purchased from the Southern Medical University Laboratory Animal Center. All animal procedures were approved by the Institutional Animal Care and Use Committee (IACUC) at Southern Medical University (L2018317).

### 4.2. Anagen Induction Assay

Paus’ method was adapted to induce vibrissae follicle anagen by depilation [42]. C57BL/6 mice were anesthetized with an intraperitoneal injection of 2% pentobarbital sodium (Sigma, St. Louis, MO, USA; *w*/*v*; 0.01 mL/g body mass). The whisker pads of mice in the telogen phase, identified by the pink skin color, were depilated using a honey and wax mixture with clippers. After depilation, the mice were randomly divided into two groups of five mice each, and 10 ng of Activin B in 1 mL of phosphate buffered solution (PBS) was administered on the whisker pads. A quantity of 1 ml of PBS was used in the control groups. These treatments were applied to the whisker pads three times a day for 20 days. The skin over the vibrissae was observed and photographed every day. At the conclusion of the treatment, mice were sacrificed, and the whisker pads were carefully dissected and fixed in 4% paraformaldehyde. 

### 4.3. Histological Analysis

The samples were dehydrated with a graded ethanol series and embedded in paraffin. Five-micrometer sections were cut for hematoxylin and eosin (H&E) staining. H&E staining was used to assess the hair cycle phase of the vibrissae follicles. For the quantitative evaluation of the anagen stage of hair follicles. Twenty longitudinal follicles per group were counted to calculate the hair cycle score as described in previous studies [30].

### 4.4. Vibrissae Follicle Organ Culture

An in vitro vibrissae culture system was established as described previously [43]. The whisker pads were cut from the mice, and the whole vibrissae follicles were dissected under a dissection microscope. Forty anagen vibrissae were collected from eight whisker pads from four mice. All isolated vibrissae follicles were randomly divided into two groups, with 20 vibrissae in each group. Vibrissae in the control group were cultured in the basal medium (Williams E medium (Gibco, Grand Island, NY, USA) containing 10 ng/mL hydrocortisone, 10 mg/mL insulin, 2 mM L-glutamine, and 100 U/mL penicillin) at 37 °C in a 5% CO_2_ atmosphere. Vibrissae in the Activin B group were cultured in basal medium supplemented with 10 ng/mL Activin B. The medium was changed every other day. Pictures were taken on 1, 3, 6, 9, and 12 days after culture. The elongation of the hair shaft was measured using Image-Pro Plus software (version 6.0, Media Cybernetics, Bethesda, MD, USA).

### 4.5. Cell Culture

Human hair germinal matrix cells (HHGMCs; Catalog#2410) were purchased from ScienCell Research Laboratories (San Diego, CA, USA). The cells were cultured in a complete growth medium (mesenchymal stem cell medium (MSCM), supplemented with 5% fetal bovine serum (FBS), 1% stem cell growth supplement (MSCGS), and 1% penicillin/streptomycin solution (P/S) (all from ScienCell, San Diego, CA, USA) and incubated at 37 °C in a 5% CO_2_ humidified atmosphere. The medium was changed every 1–2 days.

### 4.6. The Inhibitor Treatment

The cells were serum starved for 24 h and then incubated with ERK 1/2 inhibitor SCH772984 (5 μM) (Selleck, Houston, TX, USA; Catalog#S7101), P38 inhibitor SB202190 (5 μM) (Selleck, Houston, TX, USA; Catalog#S1077), or JNK inhibitor SP600125 (5 μM) (Santa Cruz, Dallas, TX, USA; CAS 129-56-6) for 2 h.

### 4.7. Cell Proliferation Assays In Vivo

After 10 ng/mL Activin B was applied to the depilated whisker pads for 10 days, EdU (5 mg/kg) was intraperitoneally injected 4 h before the mice were sacrificed. The EdU staining was conducted using a Cell-Light™ Apollo567 Stain Kit (Ribobio, Shanghai, China; C10371-1) according to the manufacturer’s protocol. Briefly, the sections of whisker pads were deparaffinized, rehydrated, and permeabilized. Then, the sections were incubated with an Apollo^®^ fluorescent reaction cocktail for 30 min. After washing, the sections were counterstained with Hoechst 33342 (1:100; Ribobio, Shanghai, China; C00033) and examined using a confocal fluorescence microscope (LSM 880; Carl Zeiss, Jena, Germany).

### 4.8. Cell Proliferation Assays In Vitro

Cell proliferation of HHGMCs was detected by Cell Counting Kit-8 (CCK-8) assay and EdU incorporation assay.

The CCK-8 (Bimake, Houston, TX, USA; B34304) assay was performed according to the manufacturer′s instructions. The cells at a concentration of 5 × 10^3^ per well were seeded into 96-well culture plates in 100 μL complete growth medium. The cells were then serum-starved for 24 h. Activin B at different concentrations (5, 10, 20, 40, 80, 160 ng/mL) was added to each well (*n* = 5 for each concentration). At 12, 24, 48, 72, and 96 h after treatment, 10 μL CCK-8 was added to each well and incubated for 2 h at 37 °C. The absorbance at 450 nm was measured by using an Epoch Microplate Spectrophotometer (BioTek, Winooski, VT, USA).

The EdU incorporation assay was carried out using the Cell-Light EdU Apollo488 in vitro kit (Ribobio, Shanghai, China; C10310-3) according to the manufacturer’s instructions. The HHGMCs were seeded in 48-well plates at a density of 10^4^ cells per well and treated with PBS or 10 ng/mL Activin B for 48 h. Then the cells were incubated with 50 μM EdU for 2 h. Following fixation with 4% paraformaldehyde and permeabilization in 0.5% Triton X-100, the cells were stained with Apollo^®^ fluorescent dye. After washing with PBS, the cells were counterstained with Hoechst 33342. The EdU-positive nuclei were determined under a fluorescence microscope. The number of positive cells was counted manually in ten fields-of-view randomly selected from each well. The percentage of positive cells was calculated.

### 4.9. Cell Cycle Analysis

For cell cycle analysis, about 1 × 10^6^ treated or untreated HHGMCs were collected and fixed with 80% ice-cold ethanol for 2 h at 4 °C. After washing and centrifugation, 0.5 mL PI/RNase (BD Pharmingen, San Diego, CA, USA) was added to the cell lysis and incubated at room temperature for 15 min in the dark. The samples were then analyzed by LSRFortessa^™^ X-20 flow cytometry (BD Pharmingen, San Diego, CA, USA). The flow cytometry data were analyzed using ModFit LT 5.0 software (Verity Software House, Topsham, ME, USA).

### 4.10. Western Blot Analysis

The HHGMCs were serum-starved for 24 h and cultured in control medium with or without 10 ng/mL Activin B. At 15 min, 30 min, 2 h, or 4 h after treatment, the cells were washed with ice-cold PBS, lysed by radioimmnoprecipitation assay (RIPA) lysis buffer (KeyGEN, Jiangsu, China), and collected by centrifugation at 14,000 rpm at 4 °C for 10 min. The protein concentration was determined using a bicinchoninic acid (BCA) Protein Assay Kit (Beyotime Technology, Shanghai, China; P0010).

Equal amounts of protein samples from different groups were separated by sodium dodecyl sulfate-polyacrylamide gel electrophoresis (SDS-PAGE) and then transferred to a polyvinylidene fluoride membrane (Millipore, Bedford, MA, USA). The membranes were blocked with QuickBlock^™^ Blocking Buffer (Beyotime, Shanghai, China; P0252) for 1 h at room temperature and then incubated with a primary antibody against p-JNK(1:1000; Cell Signaling, Danvers, MA, USA; #4668T), p-ERK(1:1000; Cell Signaling, Danvers, MA, USA; #4370S), p-P38(1:1000; Cell Signaling, Danvers, MA, USA; #4511S), p-Elk1(1:1000; Santa Cruz, Dallas, TX, USA; sc-135646), or Cyclin D1 (1:1000; Cell Signaling, Danvers, MA, USA; #2922S) overnight at 4 °C. After being washed with Tris-buffered saline containing Tween-20 (TBST), the membranes were incubated with the corresponding horseradish peroxidase (HRP)-conjugated secondary antibody (1:2000; Cwbio, Beijing, China; CW0156S) for 1 h at room temperature. The membranes were washed and exposed using the enhanced chemiluminescent substrate (ECL) (Millipore, Bedford, MA, USA). The blots were then washed with stripping buffer (Millipore, Bedford, MA, USA) for 10 min at room temperature and re-probed with antibodies against total-JNK (1:1000; Cell Signaling, Danvers, MA, USA; #9258), total-ERK (1:1000; Cell Signaling, Danvers, MA, USA; #4695S), total-P38 (1:1000; Cell Signaling, Danvers, MA, USA; #9212S), or total-Elk1 (1:1000; Santa Cruz, Dallas, TX, USA; sc-65986). Then, the intensity of the band was quantified using Quantity One software (Bio-Rad, Hercules, CA, USA).

### 4.11. Statistical Analysis

Statistical analysis was performed using SPSS13.0 software (SPSS Inc., Chicago, IL, USA). Data are expressed as mean ± standard errors of the mean (SEM). The results were compared using an independent samples *t*-test. A value of *p* < 0.05 was considered statistically significant.

## Supplementary Materials

Supplementary materials can be found at http://www.mdpi.com/1422-0067/20/4/853/s1.

## Abbreviations

EdU	5-ethynyl-2′-deoxyuridine
CCK-8	Cell Counting Kit-8
HF	Hair follicle
ORS	Outer root sheath
IRS	Inner root sheath
DP	Dermal papilla
HE	Hematoxylin and eosin
TGF-β	Transforming growth factor –β
MAPK	Mitogen-activated protein kinase
EGF	Epidermal growth factor
ACT B	Activin B
HHGMCs	Human hair germinal matrix cell
PBS	Phosphate buffer solution
PI	Propidium iodide
RIPA	Radioimmnoprecipitation assay
BCA	Bicinchoninic acid
SDS-PAGE	Sodium dodecyl sulfate-polyacrylamide gel electrophoresis
TBST	Tris-buffered saline containing Tween-20
HRP	Horseradish peroxidase
ECL	Enhanced chemiluminescent
