# Supplementary material for: Activin B Stimulates Mouse Vibrissae Growth and Regulates Cell Proliferation and Cell Cycle Progression of Hair Matrix Cells through ERK Signaling
Source: Int J Mol Sci. 2019 Feb 15;20(4):853. doi: 10.3390/ijms20040853 (PMC6413065; doi:10.3390/ijms20040853)
Supplement: Supplementary file 1 [file ijms-20-00853-s001.pdf]

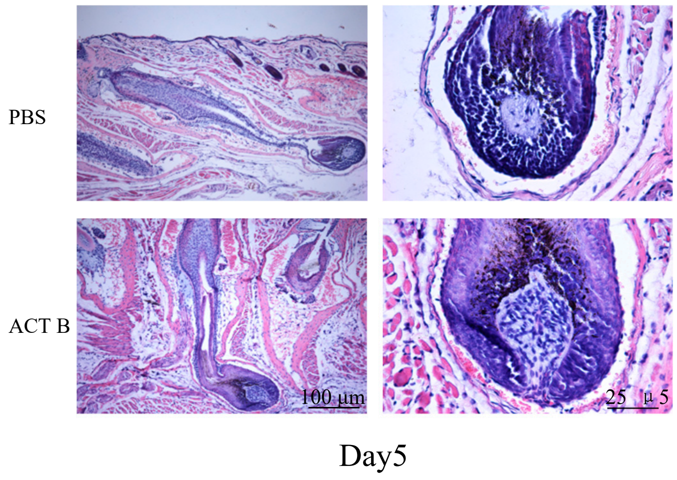

Figure S1. Representative H&E staining images of vibrissae follicles at 5 days after treatment with PBS or 10 ng/ml Activin B.

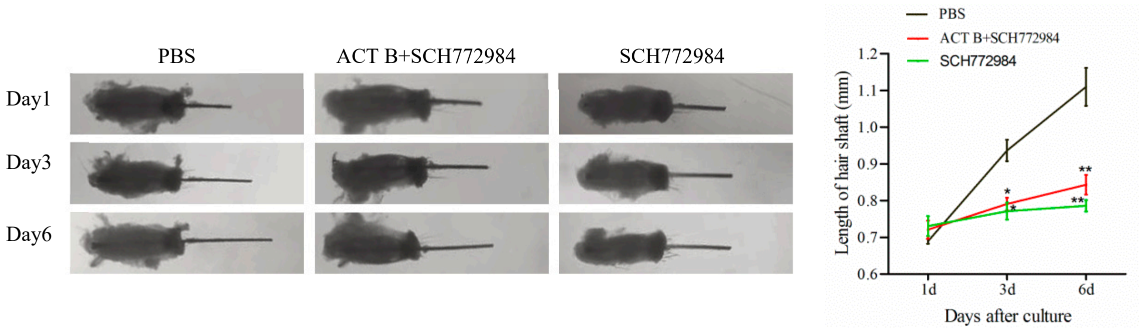

Figure S2. ERK inhibition blocks vibrissae growth. Sixty anagen vibrissae were collected from six mice. All isolated vibrissae follicles were randomly divided into three groups, with 20 vibrissae in each group. (A) Representative pictures of vibrissae cultured in the PBS, ACT B+SCH772984, and SCH772984 groups. Pictures were taken on 1, 3 and 6 days after culture. (B) Growth curve of the vibrissal shafts over time for the three groups. *$p < 0.05$; **$p < 0.01$, compared with the PBS group. Three independent experiments were conducted per data point. All error bars indicate SEM.

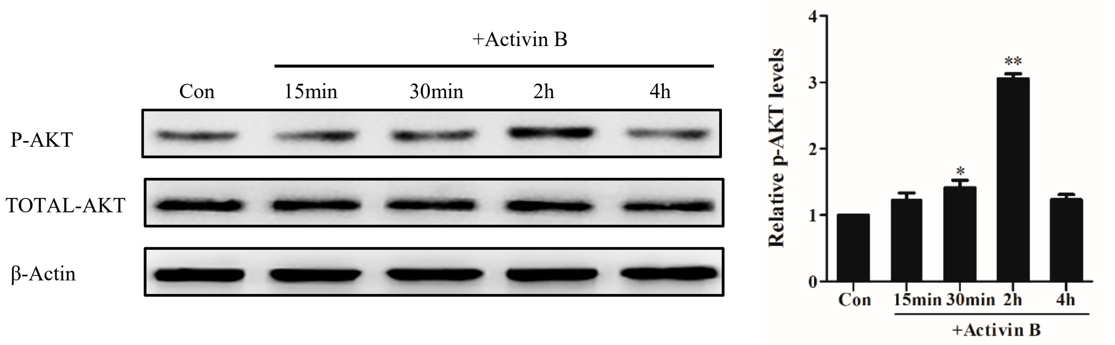

Figure S3. Activin B stimulated AKT phosphorylation in HHGMCs. The plot and the relative quantification of the expression of phosphorylation AKT in HHGMCs treated with 10 ng/ml Activin B for 15 min, 30min, 2 h, and 4 h. *p < 0.05; **p < 0.01, compared with the PBS group. Three independent experiments were conducted per data point. All error bars indicate SEM.
